# Bis(2,2′-bi-1*H*-imidazole-κ^2^ *N* ^3^,*N* ^3′^)bis­(4-methyl­benzoato-κ*O*)copper(II)

**DOI:** 10.1107/S1600536809044262

**Published:** 2009-11-04

**Authors:** Zhou Hui

**Affiliations:** a College of Chemistry and Chemical Engineering, Henan University, Kaifeng 475001, People’s Republic of China

## Abstract

In title compound, [Cu(C_8_H_7_O_2_)_2_(C_6_H_6_N_4_)_2_], the Cu^II^ atom (site symmetry 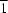) is coordinated by two *N*,*N*′-bidentate 2,2′-biimidazole ligands and two weakly bonded 4-methyl­benzoate anions, resulting in a strongly elongated *trans*-CuO_2_N_4_ octa­hedral geometry. In the crystal, adjacent mol­ecules are linked *via* pairs of N—H⋯O hydrogen bonds into chains propagating in [010].

## Related literature

For a related structure, see: Yang *et al.* (2009 ▶). 
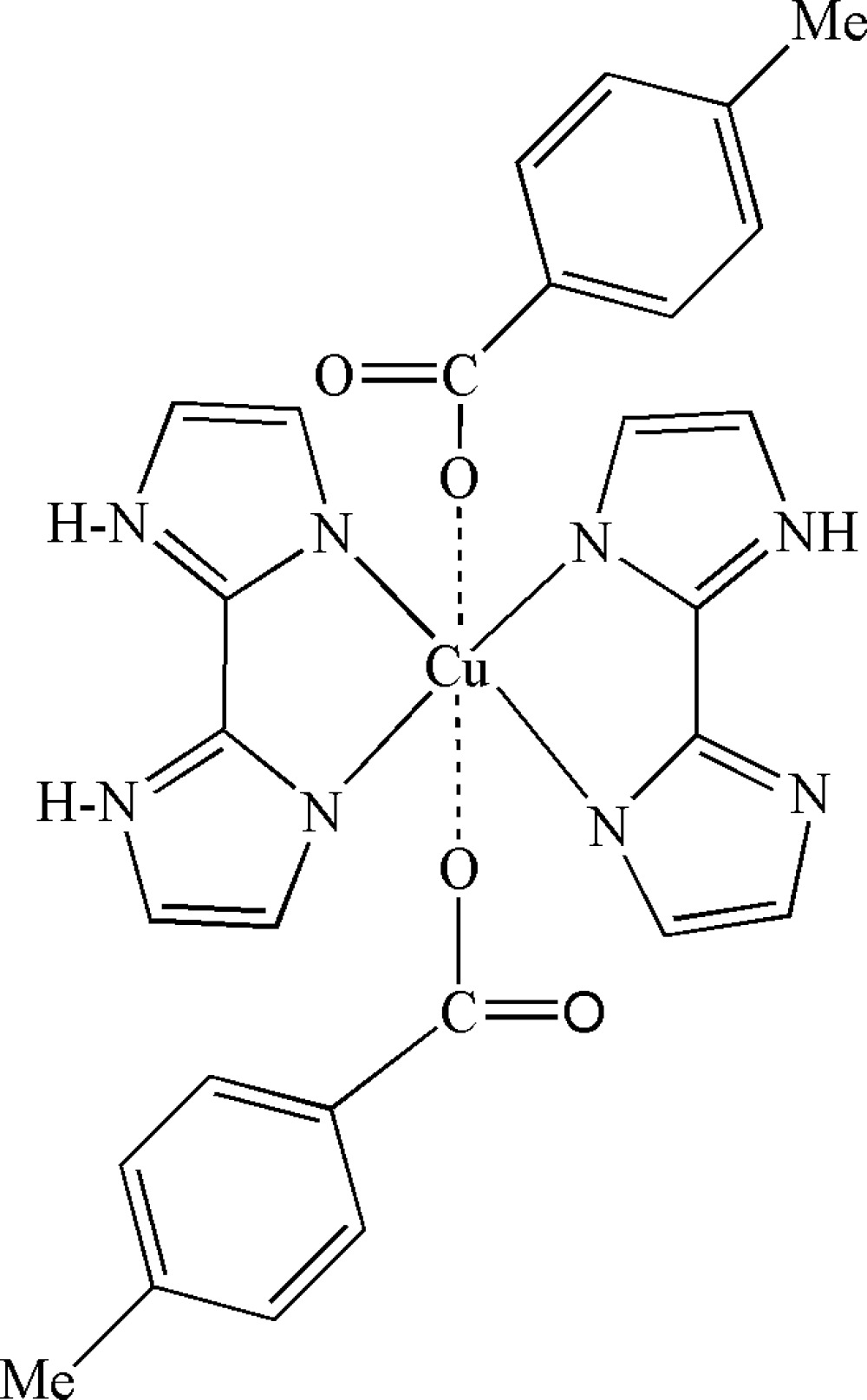


## Experimental

### 

#### Crystal data

[Cu(C_8_H_7_O_2_)_2_(C_6_H_6_N_4_)_2_]
*M* *_r_* = 602.11
Monoclinic, 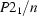

*a* = 12.2839 (9) Å
*b* = 7.3150 (5) Å
*c* = 14.9755 (11) Å
β = 93.673 (1)°
*V* = 1342.89 (17) Å^3^
*Z* = 2
Mo *K*α radiation
μ = 0.87 mm^−1^
*T* = 296 K
0.25 × 0.18 × 0.15 mm

#### Data collection

Bruker SMART APEX CCD diffractometer
Absorption correction: multi-scan (*SADABS*; Bruker, 2001 ▶) *T*_min_ = 0.813, *T*_max_ = 0.881
6459 measured reflections
2611 independent reflections
1807 reflections with *I* > 2σ(*I*)
*R*_int_ = 0.043

#### Refinement

*R*[*F* ^2^ > 2σ(*F* ^2^)] = 0.045
*wR*(*F* ^2^) = 0.104
*S* = 1.02
2611 reflections
196 parameters
2 restraints
H atoms treated by a mixture of independent and constrained refinement
Δρ_max_ = 0.26 e Å^−3^
Δρ_min_ = −0.29 e Å^−3^

### 

Data collection: *SMART* (Bruker, 2001 ▶); cell refinement: *SAINT* (Bruker, 2001 ▶); data reduction: *SAINT*; program(s) used to solve structure: *SHELXS97* (Sheldrick, 2008 ▶); program(s) used to refine structure: *SHELXL97* (Sheldrick, 2008 ▶); molecular graphics: *PLATON* (Spek, 2009 ▶); software used to prepare material for publication: *PLATON*.

## Supplementary Material

Crystal structure: contains datablocks global, I. DOI: 10.1107/S1600536809044262/hb5128sup1.cif

Structure factors: contains datablocks I. DOI: 10.1107/S1600536809044262/hb5128Isup2.hkl

Additional supplementary materials:  crystallographic information; 3D view; checkCIF report

## Figures and Tables

**Table 1:** Selected bond lengths (Å)

Cu1—N3	2.012 (2)
Cu1—N1	2.021 (2)
Cu1—O2	2.685 (2)

**Table 2:** Hydrogen-bond geometry (Å, °)

*D*—H⋯*A*	*D*—H	H⋯*A*	*D*⋯*A*	*D*—H⋯*A*
N4—H4*A*⋯O1^i^	0.890 (10)	1.755 (11)	2.644 (3)	178 (3)
N2—H2*A*⋯O2^i^	0.894 (10)	1.755 (11)	2.647 (3)	176 (3)

Symmetry code: (i) 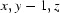.

**Fig. 1.**
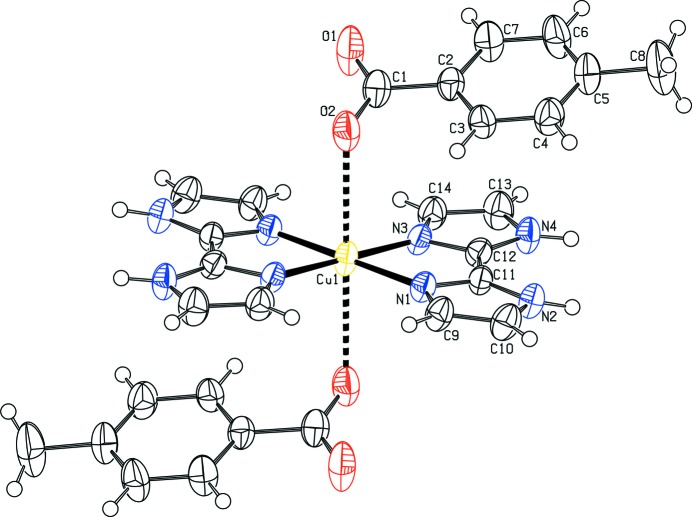
The molecular structure of (I), with displacement ellipsoids drawn at the 30% probability level. H-bonds are shown as dashed lines. Unlabeled atoms are related to labeled atoms by the symmetry transformation (2 - x, 2 - y, -z).

**Fig. 2.**
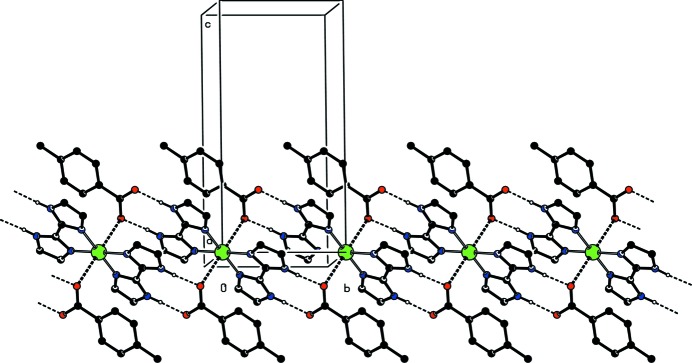
Part of the crystal structure of (I), showing the formation of the one-dimensional chain along the [010] direction. Weak contacts and hydrogen bonds are shown as thick and thin dashed lines, respectively. H atoms not involved in the hydrogen bonds have been omitted for the clarity.

## supplementary crystallographic information

### Comment

2,2'-Biimidazole is an interesting ligand because it has two N atoms and two –NH donors. Both N-donors having the stronger coordination ability and flexible coordination modes. Additionally, two –NH donors can interact with other hydrogen bonding acceptors *via* hydrogen bonds (Yang *et al.*, 2009). Herein, we report the title compound of bis(4-methyl-benzenecarboxylate-*kN*)bis(2,2'-biimidazole-*N,N*') copper, (I).

In the symmetric unit of (I), Cu^2+^ having an inversion centre was chelated by two 2,2'-biimidazole ligands through and weakly interacts with two 4-methyl benzenecarboxylate ligands acting as monodentate, which results in a elongated octahedron (Cu1—N1 2.021 (2) Å; Cu1—N3 2.012 (2) Å; Cu1—O2 2.685 (2) Å). Adjacent two Cu(C_8_H_7_O_2_)(C_6_H_3_N_4_) units are linked together by two pairs of N—H···O hydrogen bonds forming two *R*_2_^2^(9) motifs, which are further arranged into a one-dimensional chain along the [010] direction.

### Experimental

CuCl_2_.2H_2_O (0.17 g, 1.0 mmol) was added into an aqueous solution (15 ml) of 4-methyl-benzenecarboxylic acid (0.14 g, 1.0 mmol) and NaOH (0.04 g, 1.0 mmol) and refluxed for 30 min. Then an ethanol solution (10 ml) containing 2,2'-biimidazole (0.13 g, 1.0 mmol) was slowly added with continuous stirring. The resulting solution was refluxed for 3 h, filtered and kept for crystallization. After nine days, blue blocks of (I) were obtained.

### Refinement

H atoms bonded to N are located in a difference maps and refined isotropically 0.89 (1) for N—H using *DFIX* commands. All the remaining H atoms were positioned geometrically with C—H = 0.93 Å (aromatic) and 0.96 Å (methyl) and were refined as riding with *U*_iso_(H) =1.2*U*_eq_(C) (aromatic) and 1.5*U*_eq_(C) (methyl).

### Crystal data

**Table:**

[Cu(C_8_H_7_O_2_)_2_(C_6_H_6_N_4_)_2_]	*F*(000) = 622
*M**_r_* = 602.11	*D*_x_ = 1.489 Mg m^−^^3^
Monoclinic, *P*2_1_/*n*	Mo *K*α radiation, λ = 0.71073 Å
Hall symbol: -P 2yn	Cell parameters from 1035 reflections
*a* = 12.2839 (9) Å	θ = 2.7–22.3°
*b* = 7.3150 (5) Å	µ = 0.87 mm^−^^1^
*c* = 14.9755 (11) Å	*T* = 296 K
β = 93.673 (1)°	Block, blue
*V* = 1342.89 (17) Å^3^	0.25 × 0.18 × 0.15 mm
*Z* = 2	

### Data collection

**Table:**

Bruker SMART APEX CCD diffractometer	2611 independent reflections
Radiation source: fine-focus sealed tube	1807 reflections with *I* > 2σ(*I*)
graphite	*R*_int_ = 0.043
0.3° wide ω exposures scans	θ_max_ = 26.0°, θ_min_ = 2.1°
Absorption correction: multi-scan (*SADABS*; Bruker, 2001)	*h* = −13→15
*T*_min_ = 0.813, *T*_max_ = 0.881	*k* = −8→9
6459 measured reflections	*l* = −18→10

### Refinement

**Table:**

Refinement on *F*^2^	Primary atom site location: structure-invariant direct methods
Least-squares matrix: full	Secondary atom site location: difference Fourier map
*R*[*F*^2^ > 2σ(*F*^2^)] = 0.045	Hydrogen site location: inferred from neighbouring sites
*wR*(*F*^2^) = 0.104	H atoms treated by a mixture of independent and constrained refinement
*S* = 1.02	*w* = 1/[σ^2^(*F*_o_^2^) + (0.0405*P*)^2^ + 0.305*P*] where *P* = (*F*_o_^2^ + 2*F*_c_^2^)/3
2611 reflections	(Δ/σ)_max_ < 0.001
196 parameters	Δρ_max_ = 0.26 e Å^−^^3^
2 restraints	Δρ_min_ = −0.29 e Å^−^^3^

### Special details

**Table:**

Geometry. All e.s.d.'s (except the e.s.d. in the dihedral angle between two l.s. planes) are estimated using the full covariance matrix. The cell e.s.d.'s are taken into account individually in the estimation of e.s.d.'s in distances, angles and torsion angles; correlations between e.s.d.'s in cell parameters are only used when they are defined by crystal symmetry. An approximate (isotropic) treatment of cell e.s.d.'s is used for estimating e.s.d.'s involving l.s. planes.
Refinement. Refinement of *F*^2^ against ALL reflections. The weighted *R*-factor *wR* and goodness of fit *S* are based on *F*^2^, conventional *R*-factors *R* are based on *F*, with *F* set to zero for negative *F*^2^. The threshold expression of *F*^2^ > σ(*F*^2^) is used only for calculating *R*-factors(gt) *etc*. and is not relevant to the choice of reflections for refinement. *R*-factors based on *F*^2^ are statistically about twice as large as those based on *F*, and *R*- factors based on ALL data will be even larger.

### Fractional atomic coordinates and isotropic or equivalent isotropic displacement parameters (Å^2^)

**Table:**

	*x*	*y*	*z*	*U*_iso_*/*U*_eq_	
Cu1	1.0000	1.0000	0.0000	0.03766 (19)	
N1	0.89630 (18)	0.7883 (3)	0.01223 (16)	0.0349 (6)	
N2	0.8736 (2)	0.5161 (3)	0.07173 (18)	0.0397 (6)	
H2A	0.895 (3)	0.410 (3)	0.096 (2)	0.067 (11)*	
N3	1.09622 (18)	0.8469 (3)	0.08419 (16)	0.0347 (6)	
N4	1.1064 (2)	0.5980 (4)	0.16805 (17)	0.0408 (6)	
H4A	1.087 (2)	0.494 (3)	0.193 (2)	0.053 (10)*	
C9	0.7954 (2)	0.7212 (4)	−0.0174 (2)	0.0417 (8)	
H9	0.7454	0.7807	−0.0565	0.050*	
C10	0.7809 (2)	0.5549 (4)	0.0195 (2)	0.0450 (8)	
H10	0.7195	0.4809	0.0110	0.054*	
C11	0.9395 (2)	0.6593 (4)	0.06569 (19)	0.0333 (7)	
C12	1.0464 (2)	0.6939 (4)	0.10720 (19)	0.0342 (7)	
C13	1.2008 (2)	0.6939 (4)	0.1851 (2)	0.0478 (8)	
H13	1.2587	0.6610	0.2249	0.057*	
C14	1.1944 (2)	0.8452 (4)	0.1337 (2)	0.0420 (8)	
H14	1.2481	0.9346	0.1320	0.050*	
O1	1.05155 (19)	1.2894 (3)	0.24540 (17)	0.0673 (8)	
O2	0.93115 (18)	1.1925 (3)	0.13825 (16)	0.0527 (6)	
C1	0.9823 (3)	1.1777 (4)	0.2138 (2)	0.0444 (8)	
C2	0.9573 (2)	1.0140 (4)	0.2697 (2)	0.0379 (7)	
C3	0.8761 (2)	0.8939 (4)	0.2408 (2)	0.0415 (8)	
H3	0.8384	0.9127	0.1857	0.050*	
C4	0.8499 (3)	0.7468 (5)	0.2923 (2)	0.0500 (9)	
H4	0.7947	0.6676	0.2715	0.060*	
C5	0.9039 (3)	0.7145 (5)	0.3741 (2)	0.0506 (9)	
C6	0.9860 (3)	0.8328 (5)	0.4026 (2)	0.0566 (10)	
H6	1.0242	0.8122	0.4573	0.068*	
C7	1.0128 (3)	0.9816 (4)	0.3515 (2)	0.0507 (9)	
H7	1.0683	1.0602	0.3722	0.061*	
C8	0.8718 (3)	0.5549 (6)	0.4308 (3)	0.0838 (14)	
H8A	0.8992	0.4436	0.4067	0.126*	
H8B	0.7937	0.5485	0.4307	0.126*	
H8C	0.9021	0.5710	0.4910	0.126*	

### Atomic displacement parameters (Å^2^)

**Table:**

	*U*^11^	*U*^22^	*U*^33^	*U*^12^	*U*^13^	*U*^23^
Cu1	0.0373 (3)	0.0273 (3)	0.0474 (4)	−0.0028 (2)	−0.0042 (2)	0.0112 (2)
N1	0.0373 (13)	0.0266 (13)	0.0404 (15)	0.0007 (11)	−0.0007 (11)	0.0035 (11)
N2	0.0399 (14)	0.0276 (15)	0.0512 (16)	−0.0012 (12)	−0.0003 (13)	0.0073 (12)
N3	0.0383 (13)	0.0242 (13)	0.0411 (15)	−0.0033 (11)	−0.0010 (12)	0.0036 (11)
N4	0.0411 (15)	0.0313 (15)	0.0488 (17)	−0.0016 (12)	−0.0071 (13)	0.0134 (13)
C9	0.0405 (17)	0.0315 (17)	0.052 (2)	0.0030 (14)	−0.0065 (15)	0.0054 (14)
C10	0.0370 (17)	0.0360 (18)	0.061 (2)	−0.0050 (14)	−0.0052 (16)	0.0028 (16)
C11	0.0381 (16)	0.0254 (16)	0.0364 (17)	−0.0013 (13)	0.0039 (14)	0.0040 (13)
C12	0.0375 (16)	0.0270 (16)	0.0378 (18)	0.0013 (13)	0.0007 (14)	0.0005 (13)
C13	0.0412 (18)	0.046 (2)	0.054 (2)	−0.0018 (15)	−0.0124 (16)	0.0117 (17)
C14	0.0429 (18)	0.0359 (18)	0.046 (2)	−0.0071 (14)	−0.0050 (16)	0.0042 (15)
O1	0.0665 (16)	0.0519 (16)	0.0802 (19)	−0.0196 (13)	−0.0211 (14)	0.0323 (14)
O2	0.0590 (14)	0.0433 (14)	0.0549 (15)	−0.0006 (11)	−0.0050 (12)	0.0187 (11)
C1	0.0415 (18)	0.0378 (19)	0.054 (2)	0.0051 (15)	0.0002 (17)	0.0120 (16)
C2	0.0374 (16)	0.0327 (17)	0.0437 (19)	0.0052 (14)	0.0023 (14)	0.0073 (14)
C3	0.0453 (18)	0.0395 (19)	0.0392 (19)	0.0028 (15)	−0.0010 (15)	0.0092 (15)
C4	0.0507 (19)	0.0427 (19)	0.056 (2)	−0.0127 (16)	−0.0030 (17)	0.0077 (17)
C5	0.0470 (19)	0.044 (2)	0.060 (2)	−0.0069 (16)	0.0045 (17)	0.0199 (17)
C6	0.057 (2)	0.058 (2)	0.053 (2)	−0.0043 (18)	−0.0108 (18)	0.0261 (18)
C7	0.0451 (18)	0.050 (2)	0.055 (2)	−0.0119 (16)	−0.0118 (16)	0.0160 (17)
C8	0.085 (3)	0.078 (3)	0.086 (3)	−0.029 (2)	−0.010 (2)	0.045 (3)

### Geometric parameters (Å, °)

**Table:**

Cu1—N3^i^	2.012 (2)	C13—C14	1.347 (4)
Cu1—N3	2.012 (2)	C13—H13	0.9300
Cu1—N1^i^	2.021 (2)	C14—H14	0.9300
Cu1—N1	2.021 (2)	O1—C1	1.251 (4)
Cu1—O2	2.685 (2)	O2—C1	1.263 (4)
Cu1—O2^i^	2.685 (2)	C1—C2	1.504 (4)
N1—C11	1.326 (3)	C2—C3	1.378 (4)
N1—C9	1.380 (3)	C2—C7	1.384 (4)
N2—C11	1.331 (3)	C3—C4	1.374 (4)
N2—C10	1.370 (4)	C3—H3	0.9300
N2—H2A	0.894 (10)	C4—C5	1.375 (4)
N3—C12	1.332 (3)	C4—H4	0.9300
N3—C14	1.375 (4)	C5—C6	1.376 (5)
N4—C12	1.334 (3)	C5—C8	1.510 (4)
N4—C13	1.365 (4)	C6—C7	1.382 (4)
N4—H4A	0.890 (10)	C6—H6	0.9300
C9—C10	1.353 (4)	C7—H7	0.9300
C9—H9	0.9300	C8—H8A	0.9600
C10—H10	0.9300	C8—H8B	0.9600
C11—C12	1.439 (4)	C8—H8C	0.9600
			
N3^i^—Cu1—N3	180.0	N4—C13—H13	126.3
N3^i^—Cu1—N1^i^	82.25 (9)	C13—C14—N3	109.4 (3)
N3—Cu1—N1^i^	97.75 (9)	C13—C14—H14	125.3
N3^i^—Cu1—N1	97.75 (9)	N3—C14—H14	125.3
N3—Cu1—N1	82.25 (9)	O1—C1—O2	124.8 (3)
N1^i^—Cu1—N1	180.0	O1—C1—C2	117.9 (3)
C11—N1—C9	104.9 (2)	O2—C1—C2	117.3 (3)
C11—N1—Cu1	111.68 (18)	C3—C2—C7	118.3 (3)
C9—N1—Cu1	143.3 (2)	C3—C2—C1	120.1 (3)
C11—N2—C10	106.6 (3)	C7—C2—C1	121.5 (3)
C11—N2—H2A	123 (2)	C4—C3—C2	120.9 (3)
C10—N2—H2A	129 (2)	C4—C3—H3	119.5
C12—N3—C14	104.7 (2)	C2—C3—H3	119.5
C12—N3—Cu1	111.65 (18)	C3—C4—C5	121.2 (3)
C14—N3—Cu1	143.40 (19)	C3—C4—H4	119.4
C12—N4—C13	106.5 (2)	C5—C4—H4	119.4
C12—N4—H4A	126 (2)	C4—C5—C6	118.0 (3)
C13—N4—H4A	128 (2)	C4—C5—C8	120.3 (3)
C10—C9—N1	109.2 (3)	C6—C5—C8	121.6 (3)
C10—C9—H9	125.4	C5—C6—C7	121.3 (3)
N1—C9—H9	125.4	C5—C6—H6	119.4
C9—C10—N2	107.0 (3)	C7—C6—H6	119.4
C9—C10—H10	126.5	C6—C7—C2	120.2 (3)
N2—C10—H10	126.5	C6—C7—H7	119.9
N1—C11—N2	112.3 (3)	C2—C7—H7	119.9
N1—C11—C12	117.1 (2)	C5—C8—H8A	109.5
N2—C11—C12	130.6 (3)	C5—C8—H8B	109.5
N3—C12—N4	112.1 (2)	H8A—C8—H8B	109.5
N3—C12—C11	117.2 (3)	C5—C8—H8C	109.5
N4—C12—C11	130.7 (3)	H8A—C8—H8C	109.5
C14—C13—N4	107.3 (3)	H8B—C8—H8C	109.5
C14—C13—H13	126.3		

Symmetry codes: (i) −*x*+2, −*y*+2, −*z*.

### Hydrogen-bond geometry (Å, °)

**Table:**

*D*—H···*A*	*D*—H	H···*A*	*D*···*A*	*D*—H···*A*
N4—H4A···O1^ii^	0.89 (1)	1.76 (1)	2.644 (3)	178 (3)
N2—H2A···O2^ii^	0.89 (1)	1.76 (1)	2.647 (3)	176 (3)

Symmetry codes: (ii) *x*, *y*−1, *z*.
